# Therapeutic Potential of Pectin and Its Derivatives in Chronic Diseases

**DOI:** 10.3390/molecules29040896

**Published:** 2024-02-18

**Authors:** Anathi Dambuza, Pamela Rungqu, Adebola Omowunmi Oyedeji, Gugulethu Miya, Ayodeji Oluwabunmi Oriola, Yiseyon Sunday Hosu, Opeoluwa Oyehan Oyedeji

**Affiliations:** 1 Department of Chemistry, Faculty of Science and Agriculture, University of Fort Hare, P/Bag X1314, Alice 5700, South Africa; prungqu@gmail.com; 2 Department of Chemical and Physical Sciences, Faculty of Natural Sciences, Walter Sisulu University, P/Bag X1, Mthatha 5117, South Africa; aoyedeji@wsu.ac.za (A.O.O.); gmiya@wsu.ac.za (G.M.); aoriola@wsu.ac.za (A.O.O.); 3 Department of Business Management and Economics, Faculty of Economics and Financial Sciences, Walter Sisulu University, P/Bag X1, Mthatha 5117, South Africa; yhosu@wsu.ac.za

**Keywords:** pectin, non-communicable diseases, derivatives, bioavailability, bioactivity, natural products, biological studies, clinical trials

## Abstract

Non-communicable diseases (NCDs) are described as a collection of chronic diseases that do not typically develop from an acute infection, have long-term health effects, and frequently require ongoing care and therapy. These diseases include heart disease, stroke, cancer, chronic lung disease, neurological diseases, osteoporosis, mental health disorders, etc. Known synthetic drugs for the treatment or prevention of NCDs become increasingly dangerous over time and pose high risks due to side effects such as hallucination, heart attack, liver failure, etc. As a result, scientists have had to look for other alternatives that are natural products and that are known to be less detrimental and contain useful bioactive compounds. The increasing understanding of the biological and pharmacological significance of carbohydrates has helped to raise awareness of their importance in living systems and medicine, given they play numerous biological roles. For example, pectin has been identified as a class of secondary metabolites found in medicinal plants that may play a significant role in the treatment and management of a variety of NCDs. Pectin is mainly made of homogalacturonan, which is a linear polymer composed primarily of D-galacturonic acid units (at least 65%) linked in a chain by α-(1,4)-glycosidic linkages. There are also modified pectins or derivatives that improve pectin’s bioavailability. Pectin is found in the cell walls of higher plants (pteridophytes, angiosperms, and gymnosperms), particularly in the middle lamella of the plant material. Citrus pectin is used in various industries. This article compiles information that has been available for years about the therapeutic importance of pectin in chronic diseases, different modes of pectin extraction, the chemistry of pectin, and the potency of pectin and its derivatives.

## 1. Introduction

Non-communicable diseases (NCDs) are chronic diseases that are not directly transmissible from one person to another; however, the rising number of people suffering from NCDs is a cause for concern. Over the last four decades, there has been a significant rise in the prevalence of non-communicable diseases such as cancer, cardiovascular disease, neurological disorders, diabetes, and kidney disease. These conditions have emerged as leading causes of mortality worldwide, accounting for approximately 70% of all deaths [1]. Of all NCD-related deaths, 80% occur in low- and middle-income countries. Moreover, tobacco use, unhealthy diets, the consumption of food with a high saturated fat content, and a lack of exercise are also factors affecting the onset of NCDs or can be the main cause of NCD development [2,3]. Alcohol consumption is also a major factor affecting the development of NCDs almost everywhere, and it also worsens the conditions of people who already have them [4]. Natural products have played a significant role in the pharmacological and medicinal fields due to the natural components they contain [5].

Natural products are chemical entities that play a crucial role in the field of drug discovery research. Numerous drugs that possess therapeutic and pharmacological properties have been derived from natural sources, such as plants, microbes, and animals [6]. Natural products have long been known to be effective in the treatment of cancer and other NCDs. Pectin, a polysaccharide found in natural products, has been reported to have anticancer properties, being biodegradable and less toxic than alternatives [7]. Pectin is a natural complex carbohydrate and heteropolysaccharide polymer that is found in primary lamella, middle lamella, and terrestrial plants [8]. Due to advances in the understanding of carbohydrates’ biological and pharmacological activities, a growing number of people are beginning to understand the importance of carbohydrates in both living systems and medicine. Carbohydrates are involved in several biological activities. Treatments for cardiovascular diseases often use therapeutics that involve carbohydrate-based or carbohydrate-modified treatments [9]. Therefore, the purpose of this review is to explain the significance of carbohydrate pectin in the therapeutics and pharmaceutical industries, as well as its structural characteristics and pectin analogues.

## 2. Materials and Method

This review article was conducted in May 2023 using Google Scholar, Elsevier, and Science Direct to search, identify, and download articles or research papers related to pectin or pectin derivatives from 2000 to 2023, using suitable keywords such as potency, clinical trials, homogalacturonan, galacturonan, and substituted galacturonan. Mendeley (Elsevier) was used as a reference manager. All chemical structures were drawn using the ChemDraw Ultra^®^ 8.0 Software tool, while the diagrams were created using Microsoft.

## 3. Characteristics of Pectin Polymer

### 3.1. Chemistry of Pectin

Pectin is a polymer that is found naturally in higher plants and can be classified into three categories according to the following common features: The first classification is homogalacturonan (HG). HG is also known as the “smooth region” due to its linear homopolymer of α-(1,4)-linked galacturonic acid. The second category is rhamnogalacturonan-I (RG-I), which is also known as the “hair region” since it is mostly branched and accounts for 20–30% of the pectin domain. The third category is RG-II, which accounts for about 10% of the overall pectin domain and has 12 different monosaccharides and more than 20 different types of linkages. RG-II is the most intact and complex pectin domain and contains substituted galacturonans (GS) [10]. Moreover, RG-II is a highly branched polysaccharide [11]. On the other hand, RG-I consists of a linear chain of α-(1,4)-linked to D-galacturonic acid units interrupted by α-(1,2)-linked to l-rhamnopyranosyl units. Homogalacturonan is a linear polymer composed primarily of D-galacturonic acid units (at least 65%), which are linked in a chain by α-(1,4)-glycosidic linkages. Substituted galacturonans are composed of (1→4)-linked α-D-GalpA units that are branched at O-2 by (1→5)-linked α-L-Araf and terminal α-L-Araf and α-D-GalpA units [12]. The degree of esterification (DE) refers to the proportion of esterified carboxylic acid units relative to the total carboxylic acid units present in the pectin [13]. The degree of esterification, also known as the degree of methoxylation of pectin, is determined by the index of carboxyl groups that can be esterified with methyl groups. Low-methoxy (LM) pectin has less than half of the (50%) esterified carboxyl groups, while high-methoxyl (HM) pectin has more than half of the (50%) esterified carboxyl groups [14]. The DE is very important because it has a direct influence on the pectin structure and functional properties. Determining the structure of pectin is quite challenging since it can change during processing, storage, and isolation. Pectin is known to consist mainly of D-galacturonic acid (GalA) units, which are linked together by α-(1,4)-glycosidic linkages [15].

#### 3.1.1. Chemical Structure of Pectin

**Figure 1:**
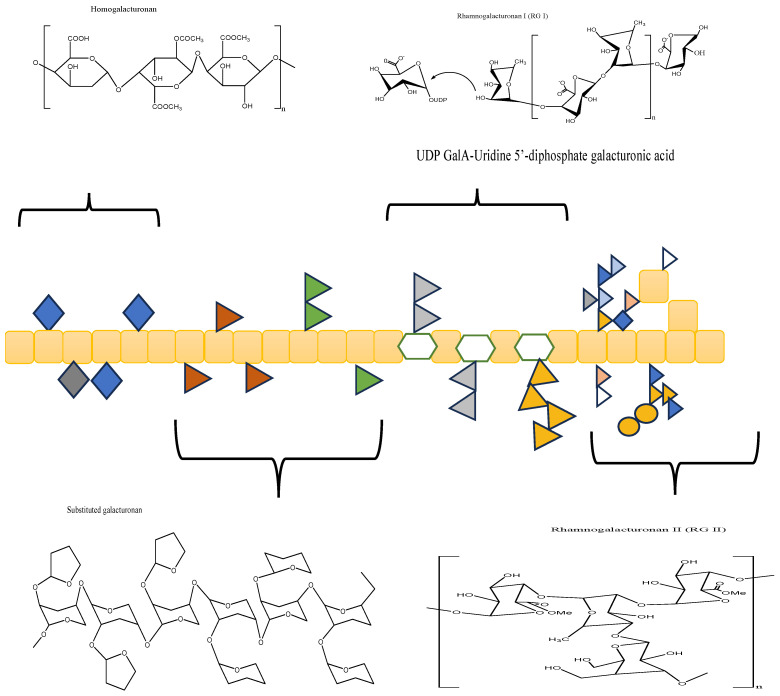

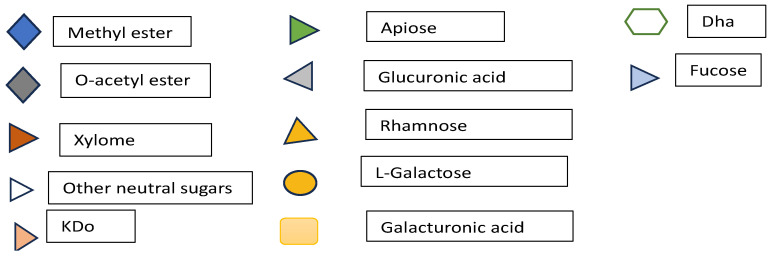
Structure of pectin.

Pectin is a complex heterocomplex structure that is considered a heteropolysaccharide since it can yield more than one type of monomer unit. From the basis of the functional aspect, it is considered an indigestible polysaccharide because it provides a layer of protection or lubrication to cells and gives plants mechanical strength [16]. Pectin has many derivatives or analogues that boost its bioavailability. Figure 1 represents the characteristic structure of pectin as a single polymer as well as its structural categories.

#### 3.1.2. Chemical Structures of Pectin Derivatives

##### Terpyridine-Metal-Pectin Derivatives

**Figure 2:**
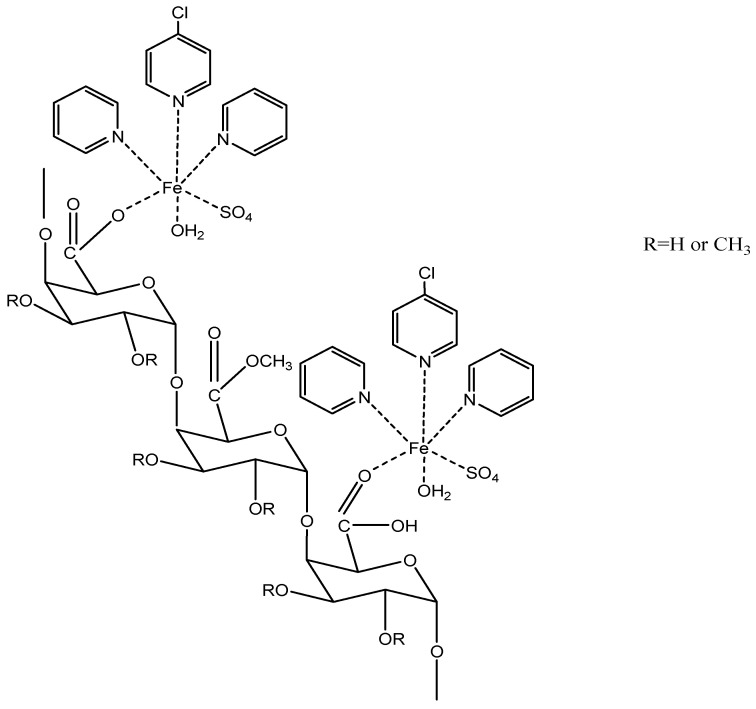
Terpyridine–iron–pectin structure.

**Figure 3:**
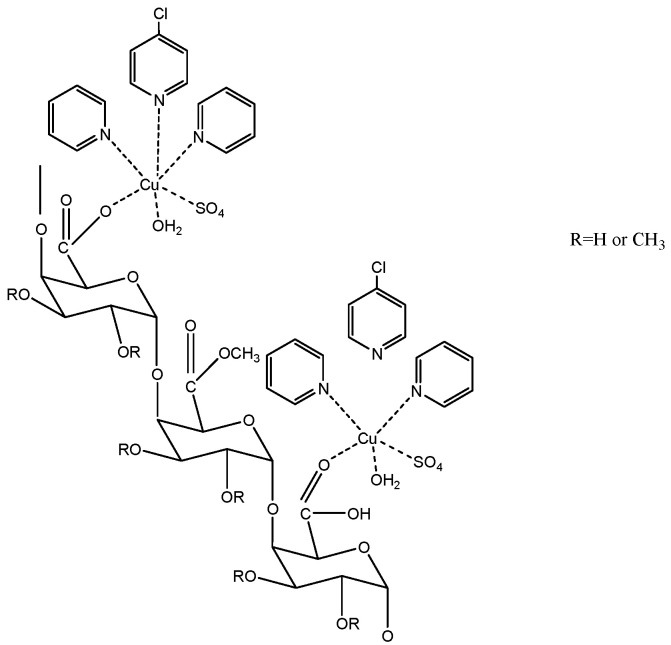
Terpyridine–copper–pectin structure.

Terpyridine-metal-pectin derivatives (see Figure 2 and Figure 3) were produced by using the following method by Hassan et al. [17]. Terpyridine-metal-pectin derivatives were produced briefly by mixing aqueous solutions of pectin with Tpy-Cu or Tpy-Cu adducts at 50 °C for 1 h. After neutralising the mixture, 75 mL of ethyl alcohol was added to precipitate the terpyridine-pectin derivatives. The derivatives were cleaned by washing them in a 3:1 volume ratio of ethyl alcohol and water until the filtrate was clear. The purified derivatives were dried for 18 h at 45 °C in a vacuum oven. These derivatives have been proven to have antimicrobial and emulsification properties.

##### Pectin Zirconium (IV) Selenotungstophosphate

**Figure 4:**
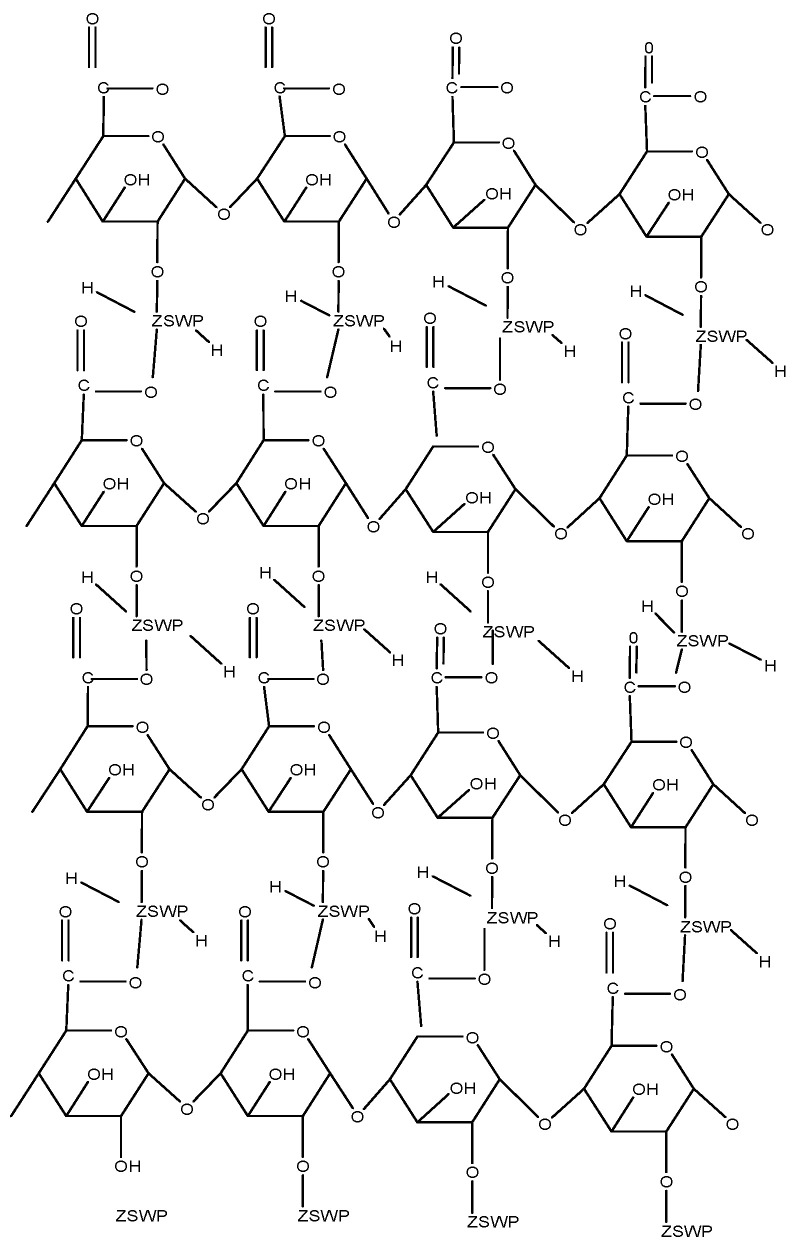
Pectin Zirconium (IV) selenotungstophosphate structure, where ZSWP is Zirconium (IV) selenotungstophosphate.

The Zirconium (IV) selenotungstophosphate (ZSWP) precipitate was produced by continuously swirling 0.1 M sodium selenite, 0.1 M sodium tungstate, and 0.1 M H_3_PO_3_ into 0.1 M zirconium oxychloride. To maintain the required pH of 1, the pH was changed using solutions of 1 M nitric M ammonia. Pectin gel was added, and the reaction mixture was stirred for 4 h at 80 °C. The formed acid or precipitates were stored for 24 h before being digested. After the supernatant liquid was decanted, the gels were filtered under suction and rinsed with distilled water. The ion exchange material was converted into H^+^ form when it was treated with 0.1 M HNO_3_ for 24 h. Pectin Zirconium (IV) selenotungstophosphate (Pc/ZSWP) was washed with distilled water and dried at 50 °C [18]. This pectin derivative was proven to possess antimicrobial activities. The structure of this pectin derivative is shown in Figure 4.

##### Pectin-Maleated Derivatives

**Figure 5:**
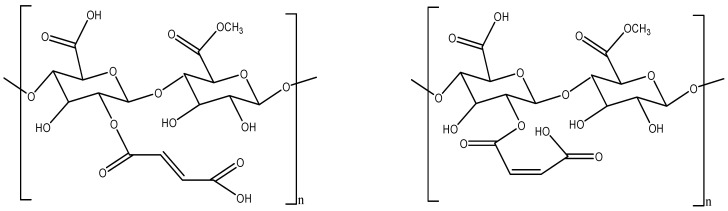
Pectin-maleated derivatives (Pec-MA) (isomers).

Pectin-maleated derivatives were formed by reacting pectin with maleic anhydride in the presence of DMF at 70 °C. Moreover, the cytotoxic effect of this derivate Pec-MA on Caco-2- cells significantly inhibited the growth of colon cancer [19]. The structures of pectin maleated derivatives are shown in Figure 5.

##### Quaternary Ammonium Derivative of Pectin

**Figure 6:**
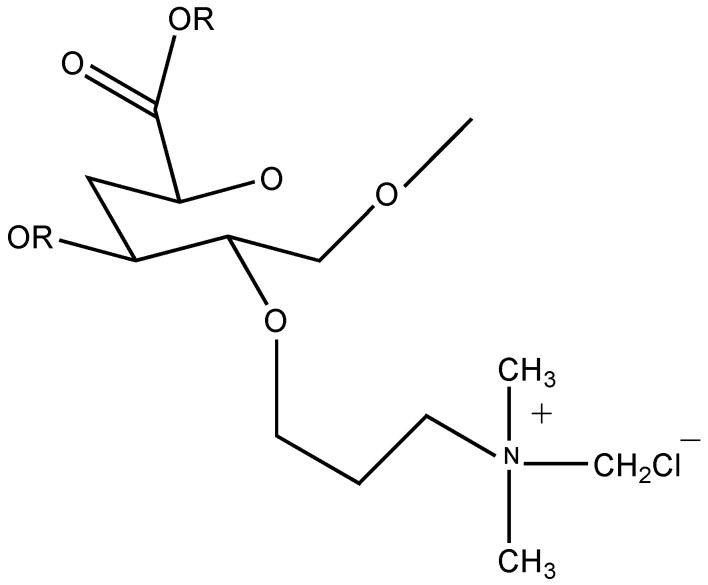
Quaternary ammonium derivative of pectin (QP) [20].

Following the method of Fan et al. [20], a quaternary ammonium derivative of pectin (QP) was obtained by using CHPTAC (3-Chloro-2-Hydroxypropyltrimethylammonium Chloride) as the etherifying agent. Also, QP showed an inhibitory effect against the three bacteria that were assessed on the strains in their investigation. Therefore, this proved that QP possesses an antimicrobial activity. The structure of quaternary ammonium derivative of pectin is shown in Figure 6.

## 4. Sources of Pectin

Pectin is a class of complex polysaccharides found in the cell walls of higher plants, where it functions as a hydrating agent and cementing material for the cellulosic network. It is particularly common in the plant’s middle lamella [21]. Soft crops or fruits are known to have a higher moisture content; this polymer is mostly found in soft plants. The firmness of the cell wall depends on how pectin is arranged. Citrus pectin is estimated to be in abundance of 20–30%, exclusively in peels [7,22]. The most profitable sources of pectin extraction are citrus fruits, apples, and by-products from their processing [23]. Sugar beet pectin (SBP), which is produced from sugar beet, is one of the sources of pectin. It has received considerable attention internationally and is known to have a lower ability to generate gels than apple pectin and citrus pectin. This is due to the presence of more acetyl groups, which prevent the bonding of the chains of pectin [22]. Apple pomace is primarily composed of skin stems (1%), seeds (24%), and 95% skin and flesh and is considered a valuable source of pectin [24].

## 5. Biological Potency of Pectin

Biological activity is a measure of the magnitude of the impact that a particular medication or extract can exert on the body or a specific system [25]. The DPPH scavenging activity assay is a widely employed method for evaluating the radical scavenging capabilities of antioxidants. This assay is used to assess the potency of a drug by comparing its potency to that of ascorbic acid, a reference compound with known antioxidant properties [26]. According to the study conducted by Sun et al. [27], pectin is effective at inhibiting free radicals; the study investigated the antioxidant properties of different forms of hawthorn pectin, namely Hydrochloric acid hawthorn pectin (HA HP), citric acid hawthorn pectin (CA HP), Cellulase hawthorn pectin (E HP), and microwave hawthorn pectin (MH HP). The IC_50_ values obtained for these pectin variants were 2.63, 2.10, 2.24, and 3.11 mg/mL, respectively. In comparison, the reference compound, ascorbic acid, exhibited an IC_50_ value of 0.01 mg/mL. Although there is a disparity in the inhibitory concentrations between pectin and ascorbic acid, it is noteworthy that pectin can effectively inhibit free radicals at higher doses [27]. An alternative in vitro approach for assessing the antioxidant properties of a compound or drug is the ABTS radical method. In a study conducted by Liu et al. [28], the inhibitory concentrations of pectin from different cultivars were evaluated, and the results revealed that *Shatangju* pectin (CPP-6) exhibited an IC_50_ value of 2.48 mg/mL, while *Xuecheng* pectin (CPP-7) had an IC_50_ value of 2.06 mg/mL. In comparison, ascorbic acid demonstrated an IC_50_ value of 31.11 μg/mL. These findings suggested that *Shatangju* pectin and *Xuecheng* pectin could potentially serve as natural antioxidants with potential applications in the pharmaceutical industry. Pectin/modified pectin has been reported to possess many pharmacological properties, such as being antiapoptosis, antiviral, and antitumor [29,30,31].

## 6. Extraction of Pectin

Extraction of pectin from plant material can be achieved using certain protocol. Table 1 below is a few of the list of methods used in pectin extraction.

## 7. Biological Activities

### 7.1. Anticancer Activity (Colon and Breast Cancer)

Cancer is a chronic disease that is mostly brought on by the body’s cells multiplying uncontrollably, spreading to other body regions, and causing tumours that may or may not be cancerous [38]. Cancer is the leading cause of death worldwide. Chemoprevention is a popular option since it prevents cancer from spreading before it has a chance to invade, which is advantageous because many cancers are dangerously contagious and difficult to treat [39]. Nevertheless, pectin seems to be the most promising biocompatible natural product, as in vivo and in vitro studies have demonstrated that pectin-derived compounds inhibit cell growth and promote apoptosis [40]. As a result, modified pectin, particularly citrus pectin, is gaining popularity because more research indicates that it is highly effective in preventing the growth and spread of cancers such as breast and colon cancer. Pectin prevents colon cancer by inhibiting galectin-3 biological functions. Galectin-3 is a chimeric gene that undergoes a noncovalent homodimerization from a monomer component with a mass of approximately 30,000 Da. Tumour cells release this β-galactoside-binding protein, which is found in the cytoplasm, nucleus, and on the cell surface. Almeida et al. [19] conducted research that revealed pectin derivatives are much more effective than pure or unmodified pectin; in their research, they used modified pectin with a maleoyl group to form the pectin derivative called Pec-MA. Pec-MA significantly inhibited colon cancer growth, as indicated by its cytotoxic effect on Caco-2 cells when compared to unmodified pectin (Pec), while healthy VERO cells showed excellent biocompatibility with the Pec-MA when compared with just raw pectin. In their investigation, they concluded that Pec-MA possesses anticancer properties, and their findings open a perspective for in vivo testing.

### 7.2. Anti-Inflammatory and Immune Modulatory Activity

Inflammation is the body’s second defence system. Through this mechanism, the immune system recognises, rejects, and initiates the healing process in response to harmful and foreign stimuli such as pathogens, irritants, and damaged cells [41]. Inflammation can be either chronic or acute. Acute inflammation is short-lived and mobilises the immune system to heal injured areas; however, this review focuses on chronic inflammation. Chronic inflammation, also known as gradual, long-term inflammation, can last for years or months. The severity and duration of chronic inflammation are typically determined by the cause of the damage and the body’s ability to repair and undo the damage [42]. With the rise of chronic inflammation across the world, researchers discovered that pectin has anti-inflammatory activity. The study by Hu et al. [43] provides evidence that pectin has direct protective effects on β-cells by reducing oxidative and nitrosative stress. The protective mechanism of pectin may involve inhibiting the pro-apoptotic protein Gal-3, preventing the delivery of intracellular danger signals, and causing the translocation of Gal-3 to perinuclear membranes. Their investigation also demonstrated that pectin with a low degree of methylation (low-DM pectin) at a high concentration exhibited the highest efficiency in protecting against oxidative and inflammatory damage. Their research proved that pectin is a suitable candidate to be used in pharmaceuticals and therapeutics as an anti-inflammatory agent.

### 7.3. Antidiabetic Activity

Diabetes is a collective term for several metabolic disorders characterised by hyperglycaemia and brought on by partial or complete insulin deficiency [44]; it is a chronic disease that affects the way your body converts food into energy. Most of the food we consume is converted by the body into sugar (glucose), which is then released into the bloodstream [45]. Excessive blood sugar in the bloodstream can lead to major health issues such as heart disease, sight loss, and renal illness if cells resist insulin response [46,47]. Type 1 diabetes is reported to affect 5–10% of the population, while type 2 diabetes affects about 90–95% of the population, making it one of the biggest threats in the world [48]. The research that was conducted by Liu et al. [49] indicated that citrus pectin possessed antidiabetic properties. In their study, they used type 2 diabetes mellitus mice (T2DM), which was induced by a high-fat diet and low-dose streptozotocin (STZ). They discovered that citrus pectin decreased fasting plasma glucose (GPG) levels, improved hyperlipidemia, increased hepatic glycogen content, and improved glucose tolerance in diabetic mice. One potential functional component to reduce insulin resistance is methoxylated apple pectin in healthy-weight individuals; soybean pectin enhances insulin and glucose responses. Citrus pectin also decreased insulin resistance, which may have increased insulin sensitivity in diabetic rats by modulating the expression of essential PI3K/Akt signalling pathway proteins, suggesting an antidiabetic mechanism for citrus pectin. Methoxylated apple pectin may be used as a functional therapeutic ingredient to reduce insulin resistance [50].

### 7.4. Anti-Hypertensive Activity

Hypertension (high blood pressure) is a chronic condition that occurs when the blood pressure in the vessels is too high. Hypertension is a major public health concern in both developing and developed countries. An apple peel is a rich source of phytochemicals such as flavonoids and pectin, and they directly or indirectly benefit the cardiovascular system and help in lowering blood pressure levels [51,52]. Agarkova et al. [53] tested pumpkin pectin’s antioxidant and hypotensive effects on a functional mousse, revealing increased antioxidant activity and reduced blood pressure in hypertensive rats. Pear pectin influences the cardiovascular system. They performed their experiment on the Sprague Dawley rat, and the 2K1C hypertension model was prepared by restricting the left renal artery with a silver clip. However, Chang-Su et al. [54] conducted a comparison study and discovered that pectin decreases blood pressure, but pear pectin decreases blood pressure more than apple pectin. This proved that pectin has a direct impact on the hypertensive effect, but the potency and strength of the pectin depends on the source. These findings indicate that pectin can be used as a key ingredient in therapeutics and pharmaceutical industries.

### 7.5. Neuroprotective Activity

Neurological diseases are conditions that affect the brain, spinal cord, and all other nerves in the body. These conditions include anxiety, depression, stroke, etc. Neurological disorders are considered the greatest global health burden, and it is expected that neurological disorders will increase exponentially in low- and middle-income countries. People with this type of illness frequently require stable support due to physical and psychosocial limitations [55]. The study by Nuzzo et al. [56] demonstrated that Lemon IntegroPectin has significant neuroprotective activity in the in vitro studies conducted by positively affecting cell viability, morphology, reactive oxygen species production, and mitochondria perturbation induced by H_2_O_2_ treatment in neuronal SH-SY5Y human cells. These findings suggested that pectin has the potential to prevent or slow down the progression of neurological diseases. Cui et al. [57] reported that modified citrus pectin (MCP) blocks galectin-3, which may be mediated by preventing the activation of the NLRP3 inflammasome via the TLR4/NF-B signalling pathway in microglia, to exert neuroprotective effects in ischemic stroke. Furthermore, according to a study done by Nishikawa et al. [58], their investigation demonstrated that modified citrus pectin has the ability to block galectin-3 and prevent the disruption of the blood–brain barrier after subarachnoid haemorrhage (SAH). In their in vivo study, they used mice as test subjects, demonstrating that this disruption occurs through the activation of ERK1/2, STAT-3, and MMP-9 via the TLR4 pathway. Their findings suggested that galectin-3 could be a potential therapeutic target for treating early brain injury following SAH.

### 7.6. Anti-Hypercholesterolemic Activity

Cholesterol is integrated into cell membranes and is necessary for the synthesis of steroid hormones. In the body, cholesterol starts with acetyl CoA and acetoacetyl-CoA, which are hydrated to form 3-hydroxy-3-methylglutaryl CoA (HMG-CoA), a rate-limiting enzyme. The body eliminates extra cholesterol by excreting bile acids and free cholesterol in bile. Too much cholesterol (called low-density lipoprotein or LDL) is a problem that can lead to hypercholesterolemia, a condition that occurs when there is excess cholesterol in the diet, bile, or intestines [59,60]. Pectin improves human health by lowering serum cholesterol and controlling blood sugar levels. Moreover, pectin can be useful as a dietary supplement for treating non-insulin-dependent diabetes as well as an anti-hypercholesterolemic agent [61]. Pectin and other soluble dietary fibres (SDF) affect the glycaemic response by delaying intestinal glucose absorption, causing the pancreas to produce less insulin; lastly, reduced insulin decreases HMG-CoA activity, lowering cholesterol synthesis [62]. Pectin plays a pivotal role in modulating the synthesis of malondialdehyde (MDA) and superoxide dismutase (SOD), thereby exerting a significant influence on the amelioration of oxidative stress and the mitigation of excessive lipid accumulation in hepatocytes (liver cells) [63].

### 7.7. Anti-Alzheimer’s Diseases Activity

Alzheimer’s disease (AD) is a prevalent age-related condition that impacts many individuals globally. Currently, it has a global prevalence of over 40 million people, accounting for approximately 70% of all dementia cases [64]. Pectin polysaccharide could potentially affect Aβ42, a significant molecule involved in the development of Alzheimer’s disease. The findings by Zeng et al. [65] suggested that a specific structural pattern present in pectin may enhance its bioactivity towards Aβ42. Furthermore, a study conducted by Liu et al. [66] using mice revealed that pectin LFA03-a could potentially serve as a targeted therapeutic medication for Alzheimer’s disease. The in vivo study demonstrated promising results, indicating the potential efficacy of pectin LFA03-a as a treatment option for Alzheimer’s disease [66]. All these findings indicate that pectin is a promising natural therapeutic agent that possesses anti-Alzheimer activity.

### 7.8. Anti-Liver Diseases Activity

Liver disease encompasses a range of conditions that impact the proper functioning of the liver, leading to its impaired functionality. Liver disease is responsible for causing approximately 2 million fatalities annually on a global scale [67]. Previous studies have demonstrated that pectin could be a natural agent to fight liver diseases. In a study by Houron et al. [68], pectin demonstrated the ability to prevent liver steatosis in obese mice at a concentration of 2% (0.06g of pectin/30 g of the mouse). Their findings indicated that a lower dose of pectin may be sufficient for curative treatment of liver steatosis. The administration of pectin results in significant alterations to the composition of the intestinal microbiota (IM), and modulating the IM has the potential to prevent liver damage caused by alcohol consumption. Therefore, targeting the IM could be a novel therapeutic approach for treating alcohol-related liver disease. Although this effect has been demonstrated in rodent models, further clinical research is necessary to validate these findings [69,70].

### 7.9. Anti-Kidney Disease Activity

Kidney disease pertains to disorders that impact the structure or functionality of the kidneys, resulting in a decline in their capacity to effectively remove waste products and surplus fluids from the bloodstream. It affects about 10% of the population globally [71]. Due to growing concerns, it became necessary to consider scientific intervention. Following such, a study by Koriem et al. [72] found that injecting rats with Octylephenol (OP) led to significant decreases in various antioxidant enzymes and molecules in kidney tissues while increasing levels of oxidative stress markers. OP also affected kidney function. However, treatment with pectin, especially at higher doses, showed antioxidant and anti-apoptotic effects in kidney toxicity induced by OP, and it effectively restored the affected parameters to normal levels. These results suggested that pectin could be a therapeutic agent to fight kidney dysfunction. Another research study conducted by Bakr 2016 [73], demonstrated that pectin can remove uremic toxic substances. This was confirmed through experiments on rats where renal failure was induced using gentamicin. The results in rats with renal failure revealed significant improvements in kidney function and enhancements in various biological, biochemical, and histopathological assessments after they were fed with a pectin diet. Khotimchenko et al. [74] conducted a study to assess the effectiveness of an enterosorbent containing low-esterified pectin, activated charcoal, and polyphepan in rats with induced renal failure. Here, pectin demonstrated the highest efficacy compared to the other substances tested. Administration of pectin led to an increase in daily urine production and a decrease in levels of blood urine and creatinine in the experimental rats. These results suggested that low-esterified pectin has the potential to be used in the development of therapeutic drugs for the comprehensive treatment of patients with chronic renal failure.

### 7.10. Clinical Trials of Pectin and Its Derivatives

Several pectins have been used in clinical trials to treat a variety of medical conditions. These clinical trials serve to ensure the safety, dosage, adverse effects, and pharmacokinetic profile of that treatment. Miyazawa et al. [75] conducted a clinical study with the clinical trial ID SRCTN19787793, whereby 18 patients (children) with cerebral palsy enrolled—16 males and 2 females from different hospitals in Japan. It was noted that 12 of these patients had recurrent vomiting, 6 had a chronic cough, and 1 of the 6 patients with chronic cough had both chronic cough and laryngitis. Children were then divided into groups and were fed food with liquid pectin but with different amounts of liquid pectin, while nurses were present to record everything, such as coughing, vomiting, and other symptoms. They observed that a high-pectin-diet food reduces vomiting and that pectin liquid concentrations reduce cough scores, both of which have an impact on reducing a wheeze or cough and other respiratory symptoms. They concluded that pectin liquid may reduce vomiting, respiratory symptoms, and gastroesophageal reflux disease (GERD) in children with cerebral palsy and may be used as an alternative treatment for GERD in addition to pharmacological therapy. There were no side effects that were observed.

Many low- and middle-income countries are plagued with typhoid fever, which is an acute febrile illness caused by *Salmonella enterica serovar Typhi* (*S. Typhi*). There were predictions of 11–21 million cases of typhoid fever in 2015, along with 148,000–161,000 deaths worldwide [76]. A clinical study by Szu et al. [77] was conducted at the Clinical Center, National Institutes of Health (NIH) (Clinical Trial ID NCT00277147). In their study, they used a pectin derivative called O-acetylated pectin (OacPec) as a plant-based typhoid vaccine. The trial enrolled 25 participants, 14 women and 11 men, with a median age of 29.0 years. The participants received a single intramuscular injection (IM) of 0.5 mL of OAcPec-rEPA, containing 25 μg of polysaccharide. Due to relocation, two participants did not complete the 26-week blood draw, but there were no significant vaccine-related responses. Six hours after injection, two volunteers experienced pain and mild general muscle aches, which went away after 24 h. OacPec proved to be an effective anti-typhoid vaccine with no adverse effects that demanded medical attention or hospitalisation.

The most recent clinical trial of the pectin derivative was done by Keizman et al. [78] (Clinical trial ID NCT01681823). Keizman and colleagues conducted the clinical trial of the pectin derivative, Pectasol C, in which prostate cancer patients (BRPC-M0) were included and administered Pectasol C-modified citrus pectin (P-MCP). The patients were given 4.8 g of P-MCP three times a day. Initially, there were 59 patients in the first phase, but after six months, there were 46 patients with no disease progression, and they entered the second phase of an additional 12 months of therapy. During the process, seven patients chose to discontinue the therapy, leaving 38 patients who completed the full 18-month duration. The results of the trial indicated that 90% of the patients experienced an improvement in prostate-specific antigen doubling time (PSADT), and 85% had no disease progression. Importantly, no patients exhibited grade 3 or 4 toxicity. According to these findings, it can be concluded that P-MCP can be used in pharmaceuticals to treat prostate cancer.

### 7.11. Limitations of Pectin as a Therapeutic Agent

Since the discovery of pectin in 1825, there have been some limitations that have hampered its utilisation. Pectin has medicinal characteristics, but it is important to note that it cannot be broken down by the human digestive system; however, it is fully metabolised by the gut microbiota. This process provides a beneficial source of nutrition for the microorganisms in the gut [79]. Pectin can also impact the availability of certain nutrients, like beta-carotene, and potentially compromise the nutritional status of individuals [80]. Dietary fibres, including pectin, can potentially have negative effects on mineral absorption, such as calcium and iron in the gastrointestinal tract. This is primarily due to their ability to bind to minerals or physically trap them, which may hinder their absorption. This can be particularly concerning for individuals who are already at risk of nutrient deficiencies [81].

## 8. Conclusions

Pectin is a carbohydrate natural biopolymer that is biocompatible and less toxic, with amazing properties from gelling to food additives; however, in this review, we paid more attention to the therapeutic effects of pectin in different chronic diseases. Over the course of several decades, significant progress has been achieved in the realm of structural modifications and applications pertaining to the polysaccharide known as pectin. This polysaccharide, with its inherent properties, has demonstrated a promising potential to be used in biomedical, pharmaceutical, and therapeutic fields. However, it is worth noting that the extent of clinical research conducted on pectin, particularly in animal models, remains limited. Consequently, further research in the future is imperative to ascertain the safety and efficacy of pectin in different chronic disease scenarios. In the future, scientific progress will increasingly rely on the principles of green chemistry and the utilisation of natural products, including biopolymers. While pectin has shown potential in disease applications, further research is necessary to explore its potential in agricultural contexts. This includes investigating the use of pectin for spoilage prevention, nutrient enhancement in plants, and its potential as a fertiliser. These studies will contribute to a more comprehensive understanding of pectin’s applications and benefits in the field of agriculture.

## Figures and Tables

**Table 1:** Different modes of pectin extraction on different plant species.

Type of Extraction	Species	Plant Part Extracted	References
Electromagnetic induction (EMI)	*Citrus sinensis* × *Poncirus trifoliata*	Peels	[32]
Conventional and microwave-assisted extraction	*Beta. vulgaris* (sugar beet)	Sugar beet pulp	[33]
Ultrasonic-assisted extraction (UAE)	*Opuntia ficus indica* (prickly pear)	Cladodes	[34]
Ultrasonic microwave-assisted extraction	*Artocarpus heterophyllus* (Jackfruit)	Peels	[35]
Conventional method and microwave-assisted method	*Hylocereus polyrhizus* (red flesh dragon) *Hylocereus undatus* (white flesh dragon) *Passiflora edulis* (passion fruit)	Peels Peels Peels	[36]
Conventional extraction	*Mangifera Indica* (mango)	Peels	[37]
